# PDI‐Mediated Drp1 Redox Regulation Preserves Mitochondrial Function and Suppresses Fibrosis in Adenine‐Induced Kidney Injury by *Lactococcus lactis* subsp. *hordniae* LB‐P22

**DOI:** 10.1111/jcmm.71456

**Published:** 2026-11-13

**Authors:** Hwa‐Young Lee, Mikyung Song, Do‐Sung Kim, Myoung Ja Chung, Hyewon Lee, Geum‐Hwa Lee, Han‐Jung Chae, Jaeseok Shim, Hwangeui Cho

**Affiliations:** ^1^ Non‐Clinical Evaluation Center Biomedical Research Institute Jeonbuk National University Hospital Jeonju Republic of Korea; ^2^ Research Institute of Clinical Medicine of Jeonbuk National University‐Biomedical Research Institute of Jeonbuk National University Hospital Jeonju Republic of Korea; ^3^ R&D Center, LISCure Bio Inc. Seongnam Republic of Korea; ^4^ Department of Pathology Jeonbuk National University Medical School Jeonju Republic of Korea; ^5^ School of Pharmacy Jeonbuk National University Jeonju Republic of Korea

**Keywords:** chronic kidney disease, dynamin‐related protein 1 (Drp1), *Lactococcus lactis* subsp. *hordniae* LB‐P22, mitochondrial dynamics, oxidative stress, protein disulfide isomerase A1 (PDIA1), renal fibrosis

## Abstract

A characteristic hallmark of chronic kidney disease (CKD) is progressive renal fibrosis, for which effective therapeutic strategies remain limited. This study investigated the renoprotective effects of *Lactococcus lactis* subsp. *hordniae* LB‐P22 in an adenine‐induced murine model of renal fibrosis, focusing on protein disulfide isomerase A1 (PDIA1)‐mediated redox regulation of dynamin‐related protein 1 (Drp1) as a central mechanism controlling mitochondrial dynamics and fibrotic progression. Mice fed a 0.75% adenine diet for three weeks developed tubular injury, interstitial fibrosis, and impaired renal function. LB‐P22 (10^8^ or 10^9^ CFU) was administered orally once daily from one week before adenine feeding until sacrifice on day 28, and attenuated these changes, with statistically significant improvement observed mainly at 10^9^ CFU. LB‐P22 reduced fibrotic remodelling and pro‐inflammatory cytokine expression (TNF‐α, IL‐6, and IL‐1β), increased superoxide dismutase, catalase, and glutathione peroxidase activities, and lowered malondialdehyde. LB‐P22 also attenuated TGF‐β1/MAPK signalling and shifted the balance of mitochondrial dynamics away from fission: adenine upregulated *Dnm1l* and *Mff* and downregulated *Opa1* and *Mfn2*, and LB‐P22 partially reversed these changes. Renal PDIA1 protein and activity were reduced by adenine and increased by high‐dose LB‐P22, whereas Drp1 sulfenylation showed the opposite pattern, consistent with the reported role of PDIA1 as a thiol reductase for Drp1. These changes were accompanied by lower mitochondrial ROS. Collectively, these findings indicate that LB‐P22 attenuates adenine‐induced renal fibrosis in association with reduced inflammation, improved redox balance, and partial normalization of mitochondrial dynamics, supporting its potential as a probiotic‐based candidate for CKD.

## 1 Introduction

Fibrosis of the kidney constitutes the ultimate pathological hallmark in various chronic kidney diseases (CKD), conditions collectively burdening roughly 13% of the global population [1, 2]. Despite its substantial prevalence and healthcare burden, effective antifibrotic therapies for CKD remain unavailable, largely due to the disease's multifactorial pathogenesis and incompletely understood molecular mechanisms [3, 4, 5]. Current clinical management therefore focuses primarily on controlling associated comorbidities, including obesity, hypertension, and diabetes, rather than directly targeting fibrotic progression.

Given these limitations, alternative therapeutic strategies that modulate disease processes through indirect or systemic pathways are being actively explored. Among these, probiotics have emerged as a promising approach; when administered in adequate amounts, they confer health benefits by restoring gut microbial balance, reinforcing intestinal barrier function, and modulating immune and inflammatory responses. This therapeutic potential is particularly relevant to the kidney through the gut‐kidney axis, an emerging paradigm in nephrology that reflects the bidirectional interaction between intestinal microbiota and renal function [6, 7]. In CKD, gut microbiota dysbiosis increases uremic metabolites, such as indoxyl sulfate and p‐cresol sulfate, which promote oxidative stress, chronic inflammation, and fibrotic progression. Accordingly, probiotic supplementation has been proposed as a therapeutic strategy for kidney injury; however, the underlying molecular mechanisms, particularly those involving mitochondrial integrity and fibrotic signalling, remain incompletely understood.

Adenine, a key component of nucleic acid metabolism and a therapeutic agent for leukopenia [8], induces nephrotoxicity and renal fibrosis when excessively accumulated in the kidney [9, 10]. Accordingly, the adenine‐induced kidney injury model is widely used to investigate the molecular mechanisms underlying renal damage and fibrosis. This model recapitulates adenine phosphoribosyltransferase (APRT) deficiency in humans, leading to the formation of 2,8‐dihydroxyadenine crystals within renal tubules and subsequent fibrotic kidney injury [11, 12]. Owing to its strong clinical relevance, the adenine‐induced nephropathy model is well established as an experimental platform for studying the pathophysiology of renal fibrosis [13, 14, 15].

Accumulating evidence indicates that mitochondrial dysfunction plays a central role in renal fibrogenesis, linking excessive mitochondrial fission, oxidative stress, and aberrant profibrotic signalling [16, 17, 18]. Dynamin‐related protein 1 (Drp1), a cytosolic GTPase, is a key regulator of mitochondrial fission [19, 20], and its activity is tightly controlled by post‐translational modifications, including phosphorylation and redox‐sensitive cysteine modifications. However, the contribution of Drp1 cysteine oxidation to renal fibrotic pathology remains unclear. This redox control may be mediated by protein disulfide isomerase A1 (PDIA1), a thiol oxidoreductase that catalyses thiol redox reactions during protein folding [21, 22, 23, 24, 25] and is present not only in the endoplasmic reticulum but also in the cytosol and mitochondria [26, 27]. Although PDIA1 has been implicated in ROS generation under pathological conditions [28], its role in regulating Drp1‐dependent mitochondrial dynamics in kidney disease remains largely unexplored. We therefore hypothesized that adenine‐induced renal injury disrupts PDIA1‐mediated redox regulation of Drp1, leading to excessive mitochondrial fission, mitochondrial ROS accumulation, and fibrotic progression. Using an adenine‐induced murine model of renal fibrosis, we investigated the therapeutic efficacy of *Lactococcus lactis* subsp. *hordniae* LB‐P22 (LB‐P22) and examined whether probiotic intervention could restore mitochondrial integrity and redox homeostasis through modulation of the PDIA1–Drp1 axis and downstream TGF‐β1/MAPK signalling pathways.

## 2 Materials and Methods

### 2.1 Reagents

The Creatinine Assay Kit (#ab65340), Urea Assay Kit (#ab83362), and assay kits for superoxide dismutase (SOD, #ab65354), glutathione peroxidase (GPx; #ab102530), and malondialdehyde (MDA; #ab233471) were purchased from Abcam (Cambridge, UK). The catalase assay kit (#707002) was obtained from Cayman Chemical (Ann Arbor, MI, USA). TRIzol Reagent (#15596026) was purchased from Invitrogen (Carlsbad, CA, USA). Haematoxylin (#H3136), Eosin Y solution (#HT110116), Masson's Trichrome Stain (MT, #HT15), and Periodic Acid–Schiff (PAS, #395B) staining reagents were obtained from Sigma‐Aldrich (St. Louis, MO, USA).

### 2.2 Preparation of *Lactococcus lactis* subsp. *hordniae* LB‐P22

The probiotic LB‐P22 samples used in the animal experiments were provided by LISCure Bio Inc., Gyeonggi‐do, Republic of Korea. The LB‐P22 (Accession No. KCTC 16234BP) was isolated from kimchi, a traditional fermented food from the Republic of Korea. The isolated strain was cultured on Lactobacilli MRS agar (BD Difco, Franklin Lakes, NJ, USA; #288210) at 30°C for 48 h. After incubation, cells were collected by centrifugation (8000× *g*, 10 min, 4°C), rinsed twice with PBS, and resuspended in sterile saline. The suspension was freeze‐dried and preserved until use.

### 2.3 Animal Model and Treatment

C57BL/6J male mice, aged seven weeks, were obtained from Central Lab Animal Inc. (Seoul, Republic of Korea). The mice were acclimated for one week in a controlled environment maintained at 22°C, 55% humidity, and a 12:12‐h light/dark cycle. Post‐acclimation, the mice were divided at random into four experimental groups (*n* = 10 per group). Group 1, normal control group receiving a standard chow diet and vehicle (oral gavage, 0.1 mL/10 g body weight); Group 2, Adenine group maintained on a 0.75% adenine diet and treated with vehicle; Group 3, LB‐P22 10^8^ group maintained on a 0.75% adenine diet and supplemented with LB‐P22 at a dose of 10^8^ colony forming units (CFU); and Group 4, LB‐P22 10^9^ group maintained on a 0.75% adenine diet and administered LB‐P22 at a dose of 10^9^ CFU. Two doses of LB‐P22 (10^8^ and 10^9^ CFU) were selected to span the range typically used for probiotic interventions and to assess the dose‐dependency of its renoprotective effects. In this study, mice were orally administered LB‐P22 at doses of 10^8^ or 10^9^ CFU for 1 week prior to the induction of CKD, followed by a 3‐week co‐administration of 0.75% adenine and LB‐P22. On day 28, mice were anaesthetised via intraperitoneal injection of ketamine (50 mg/kg) combined with xylazine (10 mg/kg). All experimental protocols were approved by the Institutional Animal Care and Use Committee (IACUC) of Jeonbuk National University Hospital and conducted in accordance with ethical guidelines (approval no. JBUH‐IACUC‐2024‐12).

### 2.4 Serum Biochemistry Analysis

After clotting at 37°C for 1 h, serum was isolated from collected blood by centrifuging at 1000× *g* for 10 min at 4°C. Serum creatinine and blood urea nitrogen (BUN) were measured using commercial assay kits (Creatinine Assay Kit, #ab65340; Urea Assay Kit, #ab83362; Abcam) according to the manufacturer's instructions, and urea concentrations were converted to BUN. Serum GOT (AST) and GPT (ALT) activities were measured using commercial kits (#XSYS0016 and #XSYS0017, respectively; Erba Mannheim, Mannheim, Germany) on an XL‐200 automated clinical chemistry analyser (Erba Mannheim).

### 2.5 Sample Handling and Preparation for LC–MS/MS Assay

For LC–MS/MS analysis, 10 μL of serum was transferred into a 1.5 mL microcentrifuge tube containing 90 μL of chilled acetonitrile and vortexed for 3 min, and subjected to centrifugation at 21,130× *g* (15,000 rpm) for 15 min at approximately 22°C–25°C. Ten microliters of the collected supernatant were placed into a clean 1.5 mL microtube, diluted with 990 μL of acetonitrile, and subsequently analysed by LC–MS/MS.

### 2.6 Quantification of Serum Uremic Toxins by LC–MS/MS

To evaluate the impact of LB‐P22 on gut‐derived uremic solutes, levels of IS and pCS in serum samples were quantified employing LC–MS/MS. UHPLC analysis was performed on a Nexera X3 system (Shimadzu, Kyoto, Japan) with a Kinetex C18 column (100 × 2.1 mm, 2.6 μm, 100 Å; Phenomenex, Torrance, CA, USA) at 30°C. The mobile phase comprised solvent A (0.1% acetic acid in water) and solvent B (acetonitrile), with a programmed gradient: 90% A/10% B at 0 min, ramping to 20% B by 3 min, then to 80% B at 3.5 min and held until 5 min, followed by re‐equilibration to the initial composition at 5.1–7.5 min. The system was run at 0.5 mL/min with a 2 μL injection volume. Mass detection employed a Triple Quad 5500+ spectrometer (SCIEX, MA, USA) in negative ESI mode under MRM settings. The ion transitions and instrument parameters were: IS, m/z 212 → 80 (quantifier, DP –70 V, CE –30 V, CXP –10 V) and m/z 212 → 132 (qualifier, CE –25 V, CXP –15 V); pCS, m/z 187 → 107 (quantifier, DP –75 V, CE –30 V, CXP –15 V) and m/z 187 → 80 (qualifier, CE –25 V, CXP –10 V).

### 2.7 Histological Staining and Fibrosis Assessment

Samples of kidney were immersed in 10% neutral‐buffered formalin, converted into paraffin blocks, and cut at a thickness of 3‐μm. Histological evaluation included H&E staining for general tissue architecture, MT staining for collagen accumulation, and PAS staining for basement membrane integrity. Among these, MT was primarily applied to visualize fibrotic alterations. Tubular injury (H&E) and interstitial fibrosis (MT) were each graded on a semiquantitative scale (0, none; 1, ≤ 25%; 2, 26%–50%; 3, 51%–75%; 4, > 75% of the field) in ten randomly chosen fields per section at ×200 magnification [14].

### 2.8 Immunohistochemistry (IHC)

For immunohistochemistry, paraffin‐embedded kidney tissues were deparaffinized and rehydrated through graded ethanol. After antigen retrieval in citrate buffer (pH 6.0, 95°C, 20 min), sections were treated with 3% hydrogen peroxide for 10 min at room temperature to block endogenous peroxidase, blocked with 2.5% normal horse serum for 20 min at room temperature, and then incubated overnight at 4°C with an anti‐PGC‐1α antibody (1:200, Santa Cruz Biotechnology, Dallas, TX, USA; #sc‐518025). After washing with 0.01 M TBST (pH 7.4), sections were incubated for 30 min at room temperature with ImmPRESS HRP Horse Anti‐Mouse IgG Polymer Reagent (MP‐7452‐50, Vector Laboratories, Newark, CA, USA). Immunoreactivity was visualized with DAB (3,3′‐diaminobenzidine; Vector Laboratories, Newark, CA, USA), and haematoxylin was used as a nuclear counterstain. Sections were finally dehydrated, mounted, and examined under an EVOS M5000 light microscope (Invitrogen, Carlsbad, CA, USA).

### 2.9 Immunofluorescence and Mitochondrial Staining

Paraffin‐embedded kidney sections and frozen sections were utilized for immunofluorescence staining and mitochondrial functional assays, respectively. For immunofluorescence, primary antibodies against PGC‐1α (1:200, Santa Cruz; #sc‐518025) and TGF‐β1 (1:200, Santa Cruz; #sc‐52893) were applied to paraffin‐embedded tissue sections. After washing in 0.01 M TBST (pH 7.4), sections were incubated with an anti‐mouse IgG (whole molecule)‐FITC antibody produced in goat (1:300, Sigma‐Aldrich; #F2021) for 1 h at room temperature under light‐protected conditions. Sections were coverslipped with VECTASHIELD Antifade Mounting Medium with DAPI (#H‐1200‐10, Vector Laboratories, Newark, CA, USA), which simultaneously counterstains nuclei. Images were acquired on an EVOS M5000 imaging system (Invitrogen). Exposure, gain, and light intensity were kept identical across all groups, and at least five randomly selected fields per section were captured (*n* = 5 mice per group). Single‐channel and merged images are presented as representative fields. For mitochondrial evaluation, 3‐μm frozen tissue sections were stained with MitoTracker Deep Red (Thermo Fisher Scientific; #M22426) to visualize mitochondria and MitoSOX Red (Thermo Fisher Scientific; #M36008) to assess mitochondrial ROS. Staining was carried out according to the suppliers' guidelines, and fluorescence visualization was performed with the same imaging platform.

For dual immunofluorescence detection of phosphorylated Drp1 and mitochondria, paraffin‐embedded kidney sections were deparaffinized, rehydrated, and subjected to heat‐induced antigen retrieval in citrate buffer (pH 6.0, 95°C, 20 min). After blocking with 5% normal goat serum for 1 h at room temperature, sections were co‐incubated overnight at 4°C with a rabbit anti‐phospho‐Drp1 antibody (1:100, PA5‐64821, Invitrogen) and a mouse anti‐VDAC1 antibody (1:100, Abcam, ab14734). After washing in 0.01 M PBST (pH 7.4), sections were incubated for 1 h at room temperature in the dark with FITC‐conjugated goat anti‐rabbit IgG (1:300, Sigma‐Aldrich, F9012) and TRITC‐conjugated goat anti‐mouse IgG (1:300, Sigma‐Aldrich, T5393). Sections were coverslipped with VECTASHIELD Antifade Mounting Medium with DAPI (#H‐1200‐10, Vector Laboratories, Newark, CA, USA), which simultaneously counterstains nuclei. Images were acquired on the EVOS M5000 imaging system (Invitrogen).

### 2.10 Proximity Ligation Assay (PLA)

The in situ PLA was performed on kidney sections using the Duolink In Situ Detection Reagents (#DUO92008, Sigma‐Aldrich) according to the manufacturer's instructions to assess the interaction between PDIA1 and Drp1. Paraffin‐embedded kidney sections (3 μm) were deparaffinized, rehydrated, and subjected to heat‐induced antigen retrieval in citrate buffer (pH 6.0, 95°C, 20 min), and were then blocked with Duolink Blocking Solution for 1 h at 37°C in a humidified chamber. Sections were incubated overnight at 4°C with a rabbit anti‐PDIA1 antibody (PDI, C81H6; 1:100, Cell Signalling Technology; #3501) and a mouse anti‐Drp1 antibody (1:100, Santa Cruz Biotechnology; #sc‐271583) diluted in Duolink Antibody Diluent. After washing in Wash Buffer A, sections were incubated with Duolink In Situ PLA probe anti‐rabbit PLUS (#DUO92002) and anti‐mouse MINUS (#DUO92004) for 1 h at 37°C. Ligation was performed with Duolink Ligase for 30 min at 37°C, followed by rolling‐circle amplification with Duolink Polymerase for 100 min at 37°C using the Duolink In Situ Detection Reagent Red kit (#DUO92008). Sections were washed in Wash Buffer B and mounted in Duolink In Situ Mounting Medium with DAPI. Negative controls, in which either primary antibody was omitted, were processed in parallel in every experiment. Images were captured on the EVOS M5000 imaging system (Invitrogen). PLA signals were counted with ImageJ (NIH, Bethesda, MD, USA) in five randomly selected non‐overlapping fields per section by an investigator blinded to group allocation, and data are expressed as the number of PLA signals per cell (*n* = 5 mice per group).

### 2.11 Analysis of Ultrastructure Using Transmission Electron Microscopy (TEM)

Tissues were fixed in 2.5% glutaraldehyde, post‐fixed in 1% osmium tetroxide, dehydrated through graded ethanol, infiltrated with Embed 812 resin, and sliced into 60–80 nm ultrathin sections. The sections were treated sequentially with 2% uranyl acetate and lead citrate, dried, and visualized under a Hitachi H‐7650 TEM (Hitachi, Tokyo, Japan). TEM was employed to examine mitochondrial morphology within renal proximal tubular cells. For each group, three mice were randomly selected, and micrographs were captured at ×10,000 magnification from cortical kidney tissue. From one representative micrograph per group, eight non‐overlapping regions were randomly selected (*n* = 8 regions), and all mitochondria visible in each region were measured for cross‐sectional area and length using ImageJ software (NIH, Bethesda, MD, USA). Quantitative analyses were performed on a per‐region basis rather than per‐cell, and all evaluations were carried out by a single trained investigator using uniform criteria to minimize variability. These regions represent technical rather than biological replicates, and the morphometric comparisons are therefore exploratory.

### 2.12 Western Blotting

Homogenization of kidney tissues was carried out using SDS buffer including 1% protease and phosphatase inhibitors. Following centrifugation of the homogenates (13,000 rpm, 10 min, 4°C), protein amount was assessed employing the BCA protein assay kit [29]. Equal protein samples were subjected to 8%–12% SDS‐PAGE, transferred to PVDF membranes, blocked with 5% non‐fat milk in TBST (TBS containing 0.1% Tween‐20), and probed overnight at 4°C with primary antibodies Nox4 (1:1000, NB110‐58851, Novus Biologicals, Centennial, CO, USA), PDIA1 (PDI, C81H6; 1:1000, #3501, Cell Signalling Technology), OPA1 (1:1000, #80471, Cell Signalling Technology), p‐Drp1 (Ser616) (1:1000, PA5‐64821, Invitrogen), Drp1 (1:1000, sc‐271583, Santa Cruz), MFF (1:1000, sc‐515664, Santa Cruz), Mfn2 (1:1000, sc‐515647, Santa Cruz), α‐smooth muscle actin (α‐SMA, 1:1000, ab124964, Abcam), TGF‐β1 (1:1000, sc‐52893, Santa Cruz), phospho‐JNK (p‐JNK, 1:1000, sc‐6254, Santa Cruz), JNK (1:1000, sc‐7345, Santa Cruz), phospho‐ERK (p‐ERK, 1:1000, sc‐7383, Santa Cruz), ERK (1:1000, sc‐135900, Santa Cruz), phospho‐p38 (p‐p38, 1:1000, sc‐166182, Santa Cruz), p38 (1:1000, sc‐81621, Santa Cruz), and β‐actin (1:1000, sc‐47778, Santa Cruz). Following washes with TBST, membranes were incubated for 1 h at room temperature with HRP‐conjugated secondary antibodies: goat anti‐rabbit IgG (1:5000, Enzo Life Sciences; #ADI‐SAB‐300‐J) or goat anti‐mouse IgG (1:5000, Enzo Life Sciences; #ADI‐SAB‐100‐J), matched to the host species of each primary antibody. Protein bands were visualized using an ECL detection system (WestGlow MAX, #BWM0400; Biomax, Guri, Republic of Korea).

### 2.13 Isolation of RNA and Quantitative Gene Expression Analysis

Total RNA from kidney tissues and human cell cultures was extracted with TRIzol (Invitrogen). One microgram of RNA was reverse transcribed using the PrimeScript First Strand cDNA Synthesis Kit (Takara Bio Inc., Shiga, Japan). qRT‐PCR was performed on a QuantStudio 6 Flex Real‐Time PCR System (Applied Biosystems, Thermo Fisher Scientific, Waltham, MA, USA), and expression was normalized to *Gapdh* (mouse) or *ACTB* (human) using the 2^−ΔΔCt^ method. The mouse primer sequences used were:

- *Acta2*: forward 5′‐TGCTGACAGAGGCACCACTGAA‐3′, reverse 5′‐CAGTTGTACGTCCAGAGGCATAG‐3′
- *Col1a1*: forward 5′‐CCTCAGGGTATTGCTGGACAAC‐3′, reverse 5′‐CAGAAGGACCTTGTTTGCCAGG‐3′
- *Il1b*: forward 5′‐TGGACCTTCCAGGATGAGGACA‐3′, reverse 5′‐GTTCATCTCGGAGCCTGTAGTG‐3′
- *Tnf*: forward 5′‐GGTGCCTATGTCTCAGCCTCTT‐3′, reverse 5′‐GCCATAGAACTGATGAGAGGGAG‐3′
- *Il6*: forward 5′‐TACCACTTCACAAGTCGGAGGC‐3′, reverse 5′‐CTGCAAGTGCATCATCGTTGTTC‐3′
- *Ppargc1a*: forward 5′‐GAATCAAGCCACTACAGACAC CG‐3′, reverse 5′‐CATCCCTCTTGAGCCTTTCGTG‐3′
- *Dnm1l*: forward 5′‐GCGAACCTTAGAATCTGTGGACC‐3′, reverse 5′‐CAGGCACAAATAAAGCAGGACGG‐3′
- *Mff*: forward 5′‐CTACTCGTAGGGCTTACCAGCA‐3′, reverse 5′‐GCTCCTTCAATGGCTGCATCTAG‐3′
- *Opa1*: forward 5′‐TCTCAGCCTTGCTGTGTCAGAC‐3′, reverse 5′‐TTCCGTCTCTAGGTTAAAGCGCG‐3′
- *Mfn2*: forward 5′‐GTGGAATACGCCAGTGAGAAGC‐3′, reverse 5′‐CAACTTGCTGGCACAGATGAGC‐3′
- *Gapdh*: forward 5′‐CATCACTGCCACCCAGAAGACTG‐3′, reverse 5′‐ATGCCAGTGAGCTTCCCGTTCAG‐3′

The human primer sequences used were:

- *ACTB*: forward 5′‐TTCTACAATGAGCTGCGTGTGG‐3′, reverse 5′‐GTGTTGAAGGTCTCAAACATGAT‐3′
- *TNF*: forward 5′‐CTCTTCTGCCTGCTGCACTTTG‐3′, reverse 5′‐ATGGGCTACAGGCTTGTCACTC‐3′
- *IL6*: forward 5′‐AGACAGCCACTCACCTCTTCAG‐3′, reverse 5′‐TTCTGCCAGTGCCTCTTTGCTG‐3′

### 2.14 Measurement of SOD, Catalase, GPx, and MDA

To evaluate oxidative stress markers, frozen kidney samples were weighed and homogenized in ice‐cold saline to prepare a 10% (w/v) tissue homogenate. Superoxide dismutase (SOD), catalase, glutathione peroxidase (GPx) activities, and malondialdehyde (MDA) levels were measured using commercial assay kits according to the manufacturer's instructions.

### 2.15 NADPH Oxidase (NOX) Activity

NOX activity was measured by the lucigenin‐enhanced chemiluminescence Method [30]. Briefly, frozen renal tissues were homogenized in ice‐cold assay buffer and centrifuged to collect the supernatant. Protein concentrations were determined using a standard protein assay, and equal amounts of protein were used for each reaction. Protein samples were added to phosphate buffer containing 5 μM lucigenin (Sigma‐Aldrich) and 100 μM NADPH (Sigma‐Aldrich). Luminescence, taken as an index of NOX‐derived ROS production, was expressed as relative NOX activity compared with the control group.

### 2.16 Measurement of Intracellular Reactive Oxygen Species (ROS) Using CellROX Assay

Intracellular reactive oxygen species (ROS) levels were measured using CellROX Deep Red Reagent (Invitrogen, Thermo Fisher Scientific, C10422, Waltham, MA, USA). A 10 mM stock solution of CellROX reagent was prepared in dimethyl sulfoxide (DMSO) and diluted to final working concentration of 5 μM. Frozen kidney sections (3 μm) were incubated with the working solution for 30 min at 37°C in the dark, washed twice with phosphate‐buffered saline (PBS) to remove excess dye. Sections were coverslipped with VECTASHIELD Antifade Mounting Medium with DAPI (#H‐1200‐10, Vector Laboratories), which simultaneously counterstains nuclei. Images were acquired on the EVOS M5000 imaging system (Invitrogen, Carlsbad, CA, USA).

### 2.17 Protein Disulfide Isomerase (PDI) Activity Assay

Protein disulfide isomerase (PDI) reductase activity was measured using the PROTEOSTAT PDI Assay Kit (Enzo Life Sciences, Cat. No. ENZ‐51024, Farmingdale, NY, USA), according to the manufacturer's instructions. Tissue samples were homogenized in extraction buffer containing 50 mM potassium phosphate (pH 7.4), 300 mM potassium bromide, and a protease inhibitor cocktail. After centrifugation, the supernatants were collected, and total protein concentrations were determined using a standard protein assay. For each reaction, 10 μg of total protein was used to assess PDI reductase activity. Fluorescence signals corresponding to PDI activity were measured as specified in the kit protocol.

### 2.18 DCP‐Bio1 Assay to Detect Sulfenylated Proteins

Kidney tissues were homogenized and lysed in the presence of 200 μM DCP‐Bio1 (Kerafast, Boston, MA, USA) to biotin‐label sulfenylated (–SOH) cysteine residues, with N‐ethylmaleimide included in the lysis buffer to block free cysteine thiols. DCP‐Bio1–tagged proteins were then pulled down with streptavidin‐conjugated beads, followed by immunoblotting for Drp1 to detect Drp1 sulfenylation (Drp1‐SOH), as previously described [31].

### 2.19 Cell Culture

HEK293 (human embryonic kidney 293), a human embryonic kidney‐derived cell line, was purchased from the Korea Cell Line Bank (KCLB No. 21573, Seoul, Republic of Korea) and maintained in RPMI 1640 medium (Welgene, Daegu, Republic of Korea; #LM011‐01) without HEPES, containing 10% FBS and antibiotics (penicillin 100 U/mL and streptomycin 100 μg/mL; 1%), under conditions of 37°C and 5% CO_2_ in a humidified incubator.

### 2.20 Evaluation of HEK293 Cell Viability and Inflammatory Gene Expression Following LB‐P22 Conditioned Medium Treatment

The 1 × 10^9^ CFU/mL of LB‐P22 was maintained with 50 mL of RPMI 1640 culture medium without serum for 24 h at 30°C. LB‐P22 conditioned medium (CM; pH 7.2) was obtained post‐culture by centrifugation (685× *g*, 10 min), filtration with a 0.22 μm Millipore filter, and preservation at −80°C for later use. For the viability assay, HEK293 cells were seeded in 96‐well plates and allowed to attach for 24 h; cells were then treated with LB‐P22 CM at 0.1%, 0.5%, 1%, or 2% (v/v) in the presence or absence of 5 mM IS, with the final volume adjusted to 200 μL per well. After a further 24 h, viability was assessed using the WST‐8 cell proliferation assay (Biomax) according to the manufacturer's instructions. For gene expression analysis, cells were seeded in 6‐well plates at 3 × 10^5^ cells/well (*Tnf*) or 6 × 10^5^ cells/well (*Il6*) and treated under the same conditions in a final volume of 2 mL per well before RNA extraction.

### 2.21 Statistical Analysis

All statistical evaluations were conducted with GraphPad Prism version 10.5.0 (GraphPad Software, San Diego, CA, USA). Comparisons among groups were assessed by one‐way ANOVA with Tukey's multiple comparison test. Results are reported as mean ± SD, and values of *p* < 0.05 were considered statistically significant. Data were obtained from the following numbers of animals per group: body weight, histological analyses (H&E, Masson's trichrome, PAS), serum biochemical analyses, tissue oxidative‐stress assays (SOD, catalase, GPx, MDA), and RT‐PCR, *n* = 10; serum uremic toxins, *n* = 6; NADPH oxidase and PDI activity assays, proximity ligation assay, MitoTracker/MitoSOX, and immunofluorescence analyses, *n* = 5; transmission electron microscopy morphometry, *n* = 8 regions; and Western blot densitometry, *n* = 6 (randomly selected samples). Outliers were identified using Grubbs' test (*α* = 0.05); accordingly, one indoxyl sulfate value in the LB‐P22 10^8^ CFU group was excluded.

## 3 Results

### 3.1 LB‐P22 Attenuates Renal Injury and Fibrosis in Adenine‐Induced Kidney Injury

To investigate the therapeutic efficacy of *Lactococcus lactis* subsp. *hordniae* LB‐P22, mice received oral LB‐P22 (10^8^ or 10^9^ CFU, once daily) for one week, after which chronic kidney disease (CKD) was induced by feeding a 0.75% adenine diet for three weeks while LB‐P22 administration was continued. Mice were sacrificed on day 28 (Figure S1A). Histopathological examination of kidney tissues using haematoxylin and eosin (H&E), periodic acid‐Schiff (PAS), and Masson's trichrome (MT) staining revealed severe tubular damage, loss of tubular architecture, and marked interstitial fibrosis in adenine‐treated mice. In contrast, LB‐P22 supplementation, particularly at the dose of 10^9^ CFU, significantly attenuated these pathological alterations. Quantitative histological analysis confirmed a significant reduction in renal injury scores at 10^9^ CFU, whereas 10^8^ CFU did not reach statistical significance (Figure 1A–C). Consistent with these histological improvements, biochemical assessment demonstrated significantly elevated serum creatinine and blood urea nitrogen (BUN) levels in adenine‐treated mice, indicating impaired renal function. LB‐P22 supplementation significantly reduced serum creatinine at both doses, whereas BUN was significantly reduced only at 10^9^ CFU, reflecting partial preservation of kidney function (Figure 1D,E). Adenine‐induced body weight loss was significantly attenuated by LB‐P22 at 10^9^ CFU on day 28, but not at 10^8^ CFU (Figure S1B). With respect to hepatic safety, adenine administration did not significantly alter serum GOT or GPT, and LB‐P22 supplementation produced no adverse changes in either parameter (Figure S1C,D). To further substantiate the anti‐fibrotic effects of LB‐P22, renal expression of fibrosis‐associated markers was evaluated. Quantitative RT‐PCR analysis revealed that adenine exposure significantly increased the mRNA expression of *Acta2* (α‐SMA) and collagen type I alpha 1 (*Col1a1*), whereas LB‐P22 treatment significantly suppressed their expression at 10^9^ CFU, with no significant change at 10^8^ CFU (Figure 1F,G). These transcriptional changes were corroborated at the protein level, as immunoblot analysis demonstrated a pronounced reduction in α‐SMA protein expression in kidneys from high‐dose LB‐P22 (10^9^ CFU)‐treated mice compared with the adenine‐only group (Figure 1H,I). Collectively, these results indicate that LB‐P22 effectively attenuates adenine‐induced renal injury and fibrosis by improving renal function and suppressing fibrotic remodelling in CKD.

**FIGURE 1:**
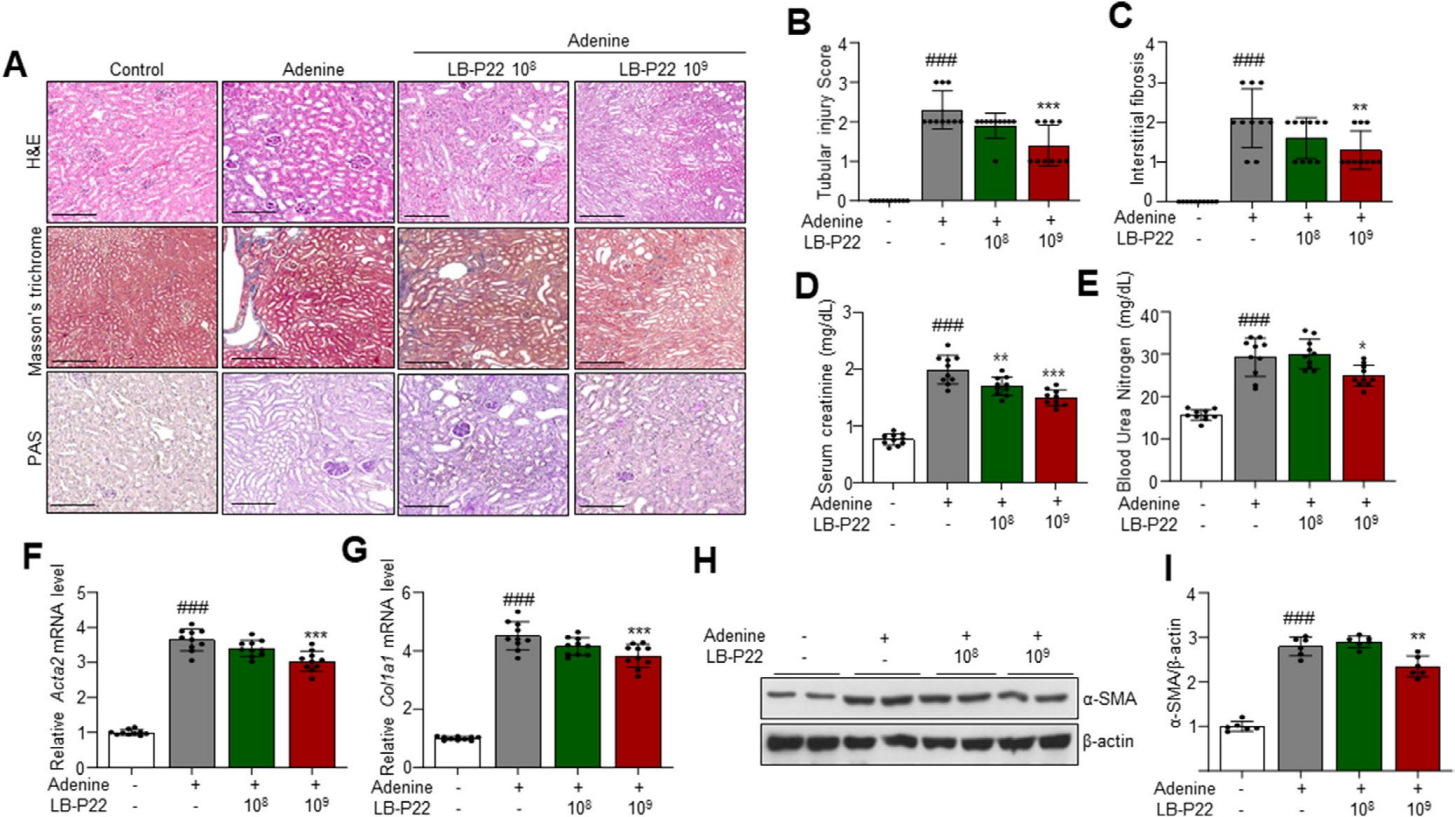
LB‐P22 ameliorates adenine‐induced renal injury and fibrosis in mice. Mice maintained on a 0.75% adenine‐supplemented diet for three weeks developed renal tubular dilation, epithelial atrophy, interstitial extracellular matrix accumulation, and impaired renal function. Oral administration of LB‐P22 (10^8^ or 10^9^ CFU) for four weeks mitigated these pathological alterations, with statistically significant improvement observed mainly at 10^9^ CFU. (A) Representative kidney sections stained with haematoxylin and eosin (H&E), Masson's trichrome (MT), and periodic acid–Schiff (PAS) to assess tubular atrophy score, fibrotic remodelling, and basement membrane integrity. Scale bar = 100 μm. (B, C) Histological scores quantifying tubular injury (H&E) and interstitial fibrosis (MT). (D, E) Serum creatinine and blood urea nitrogen (BUN) levels were measured to evaluate renal functional status. (F, G) Relative mRNA expression levels of fibrotic markers *Acta2* (α‐SMA) and *Col1a1* (type I collagen α1 chain) in renal tissues, as determined by qRT‐PCR. (H, I) Representative immunoblot images showing α‐SMA protein expression across treatment groups and corresponding quantitative analysis. β‐actin was used as a loading control for normalization. Sample sizes: *n* = 10 per group for panels A–G, and *n* = 6 for the immunoblot quantification in panels H and I. Data are presented as the mean ± SD. Statistical significance was assessed by one‐way analysis of variance (ANOVA), followed by Tukey's multiple comparisons test. ^###^ *p* < 0.001 versus the control group; *p < 0.05, **p < 0.01, and ***p < 0.001 versus the adenine‐treated group.

### 3.2 LB‐P22 Selectively Reduces Indoxyl Sulfate (IS), but Not p‐Cresol Sulfate (pCS), in Serum

CKD is frequently associated with dysbiosis of the gut microbiota, leading to enhanced production and systemic accumulation of gut‐derived uremic toxins. Among these, IS and pCS are well‐characterized protein‐bound toxins generated through the microbial metabolism of dietary tryptophan and tyrosine, respectively, in the colon [32]. Impaired renal clearance in CKD results in their accumulation, contributing to inflammation, oxidative stress, and progression of renal damage. To assess LB‐P22's influence on uremic toxins, we measured serum levels of IS and pCS. Adenine‐fed mice exhibited markedly elevated IS levels and a comparatively modest, non‐significant increase in pCS. IS levels were significantly reduced by LB‐P22 at both doses (Figure 2A). Serum pCS levels remained similar across adenine and LB‐P22 groups (Figure 2B), suggesting selective modulation of gut‐derived metabolites by LB‐P22.

**FIGURE 2:**
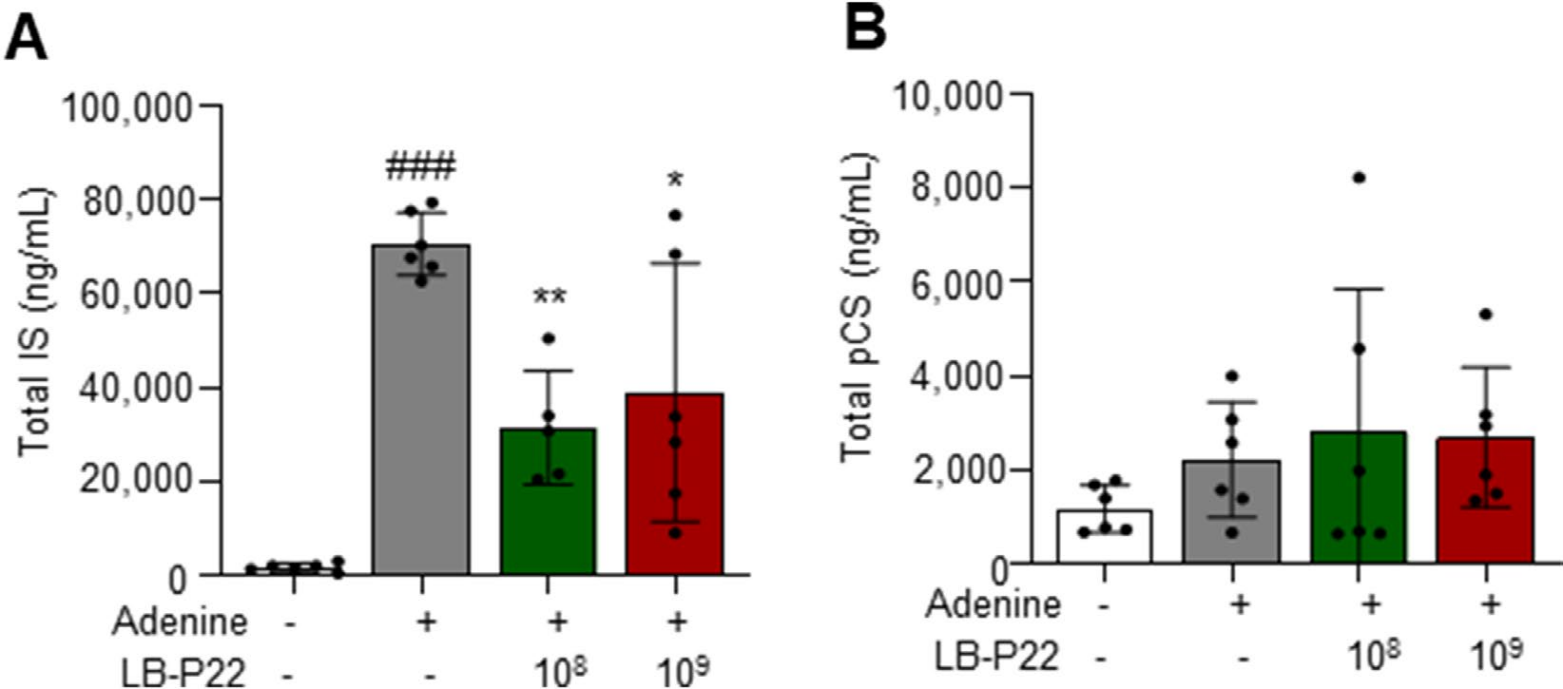
LB‐P22 selectively reduces serum IS levels in mice with adenine‐induced CKD. (A, B) Levels of IS and pCS, two gut‐derived uremic toxins, were determined in serum (*n* = 6 mice per group; one indoxyl sulfate value in the LB‐P22 10^8^ CFU group was excluded as an outlier by Grubbs' test, *α* = 0.05). Data are presented as the mean ± SD. Statistical significance was assessed by one‐way analysis of variance (ANOVA), followed by Tukey's multiple comparisons test. ^###^ *p* < 0.001 versus the control group; *p < 0.05 and **p < 0.01 versus the adenine‐treated group.

### 3.3 LB‐P22 Preserves PDIA1 Expression and Activity and Attenuates Nox4‐Dependent Oxidative Stress in Adenine‐Induced Kidney Injury

To investigate redox‐dependent mechanisms underlying adenine‐induced kidney injury, we first assessed NADPH oxidase activity and Nox4 expression in renal tissues. Adenine administration significantly increased NADPH oxidase activity compared with control kidneys, whereas LB‐P22 supplementation significantly attenuated this increase at 10^9^ CFU (Figure 3A). Consistently, immunoblot analysis revealed that adenine significantly upregulated renal Nox4 protein expression, which was significantly suppressed by LB‐P22 treatment at 10^9^ CFU (Figure 3B). In parallel, intracellular ROS levels were evaluated using CellROX staining in kidney sections. Adenine‐treated kidneys exhibited a robust increase in CellROX fluorescence intensity, indicating excessive ROS accumulation, whereas LB‐P22 supplementation visibly reduced CellROX fluorescence; these images are presented as representative, qualitative observations and were not quantified (Figure 3C). These results indicate that LB‐P22 effectively suppresses Nox4‐dependent oxidative stress in adenine‐induced renal injury.

**FIGURE 3:**
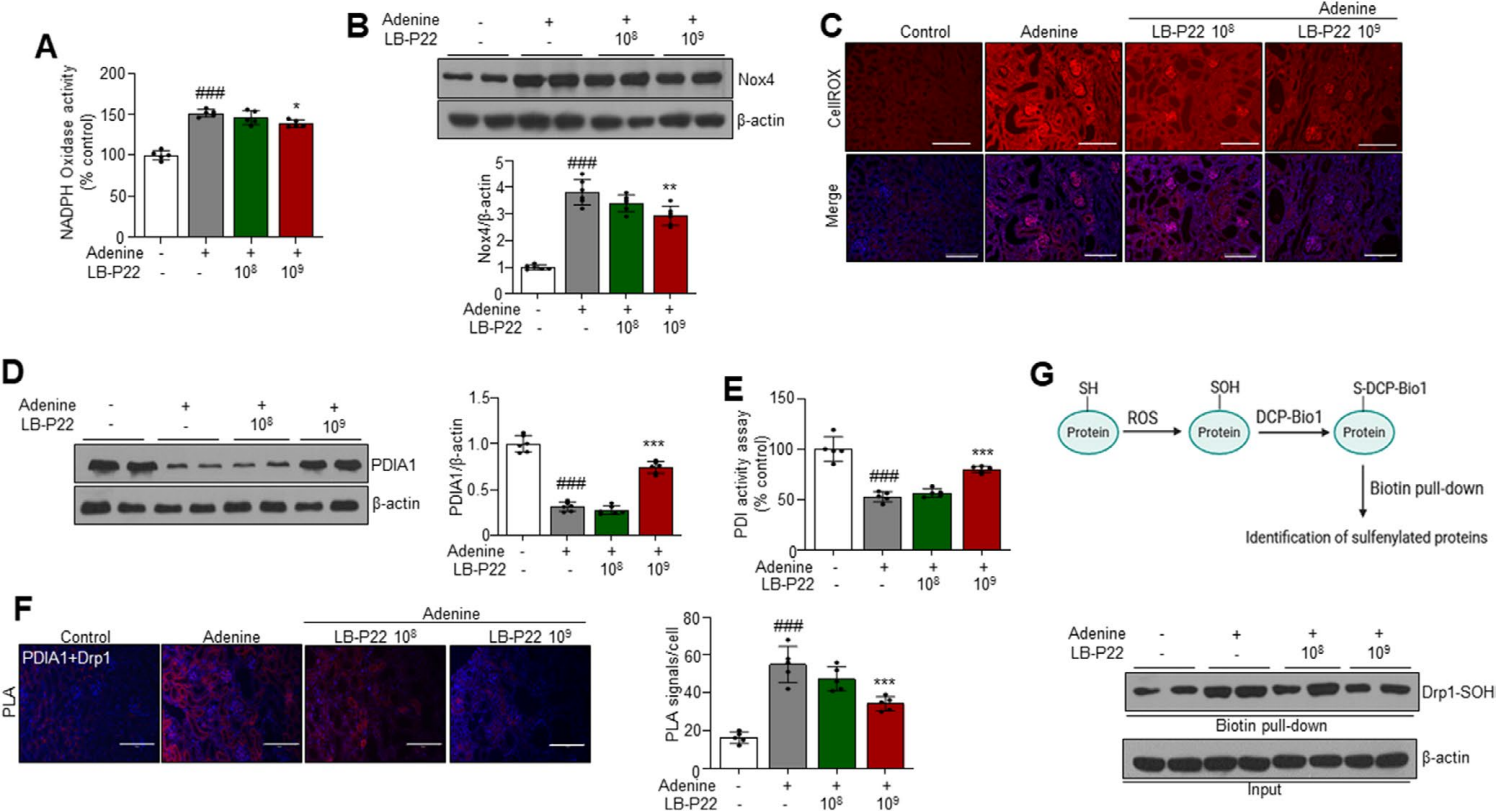
LB‐P22 preserves PDIA1 expression and activity and attenuates Nox4‐dependent oxidative stress in adenine‐induced kidney injury. (A) Renal NADPH oxidase activity measured in kidney tissues from control, adenine‐treated, and LB‐P22–supplemented mice. (B) Immunoblot analysis of Nox4 protein expression in renal tissues across experimental groups. β‐Actin was used as a loading control. (C) Representative fluorescence images of kidney sections stained with CellROX to detect intracellular reactive oxygen species (ROS) (upper panels) and the corresponding merged images with DAPI nuclear counterstaining (lower panels); these images are representative and were not quantified. Scale bar = 150 μm. (D) Immunoblot analysis of protein disulfide isomerase A1 (PDIA1) expression in renal tissues from each group. β‐Actin was used as a loading control. (E) PDIA1 enzymatic activity measured in tissue lysates and expressed as a percentage of the control group. (F) Proximity ligation assay (PLA) showing PDIA1–Drp1 interactions in kidney sections. Representative images (left) and quantitative analysis of PLA signals per cell (right) are shown. Scale bar = 150 μm. (G) Schematic illustration of the DCP‐Bio1 labelling and biotin pull‐down approach used to detect protein sulfenylation (upper). Representative immunoblot images showing Drp1 sulfenylation (Drp1‐SOH) following biotin pull‐down from tissues (lower); β‐actin from the corresponding whole‐tissue lysate (input) is shown as a loading control. These blots are representative and were not subjected to densitometric quantification. Data are presented as the mean ± SD (*n* = 5 mice per group for panels A, E, and F; *n* = 6 mice per group for panels B and D). Statistical significance was assessed by one‐way analysis of variance (ANOVA), followed by Tukey's multiple comparisons test. ^###^ *p* < 0.001 versus the control group; *p < 0.05, **p < 0.01, and ***p < 0.001 versus the adenine‐treated group.

Given the established role of protein disulfide isomerase A1 (PDIA1) as a redox‐sensitive regulator of mitochondrial dynamics, we next examined whether adenine‐induced oxidative stress alters PDIA1 expression and activity. Immunoblot analysis showed that PDIA1 protein levels in kidney tissue were significantly reduced by adenine exposure; compared with the adenine‐only group, PDIA1 expression was significantly increased in mice treated with high‐dose LB‐P22 (10^9^ CFU), whereas the low dose (10^8^ CFU) showed no significant difference (Figure 3D). Consistent with these findings, PDIA1 enzymatic activity was significantly decreased in adenine‐treated kidneys but was substantially recovered following LB‐P22 supplementation, particularly at 10^9^ CFU (Figure 3E). Under physiological conditions, PDIA1 has been reported to interact with dynamin‐related protein 1 (Drp1), thereby maintaining Drp1 in a reduced redox state. To determine whether adenine‐induced oxidative stress affects PDIA1–Drp1 interactions in vivo, we performed proximity ligation assays (PLA) in renal sections. Adenine administration significantly increased PDIA1‐Drp1 PLA signals, reflecting aberrant redox‐dependent interaction under pathological oxidative conditions. Notably, PLA signals were significantly reduced in mice treated with high‐dose LB‐P22 (10^9^ CFU) compared with the adenine‐only group, whereas the low dose showed no significant difference (Figure 3F). To directly assess oxidative modification of Drp1, we examined Drp1 sulfenylation using a DCP‐Bio1 labelling followed by biotin pull‐down. A schematic diagram illustrates the ROS‐mediated oxidation of cysteine thiol (–SH) residues to sulfenic acid (–SOH), which facilitates Drp1 activation (Figure 3G, upper). Consistent with this model, adenine administration appeared to increase Drp1‐SOH levels in renal tissues, whereas this signal appeared weaker in the LB‐P22–supplemented group; representative immunoblots are shown without densitometric quantification (Figure 3G, lower). Collectively, these results indicate that adenine‐induced kidney injury disrupts the PDIA1–Drp1 redox regulatory axis through Nox4‐driven oxidative stress. LB‐P22 supplementation restored renal redox homeostasis by suppressing Nox4‐dependent ROS production and preserving PDIA1 expression and enzymatic activity, and this was accompanied by reduced Drp1 sulfenylation in representative immunoblots.

### 3.4 LB‐P22 Inhibits Drp1‐Mediated Aberrant Mitochondrial Fission in Adenine‐Induced Kidney Injury

To determine whether LB‐P22 modulates mitochondrial dynamics disrupted by adenine‐induced kidney injury, we examined the expression of key regulators involved in mitochondrial fission and fusion in renal tissues. Quantitative RT‐PCR analysis demonstrated that adenine administration significantly upregulated the mRNA expression of the mitochondrial fission–associated genes *Dnm1l* (Drp1) and *Mff* compared with control kidneys. Compared with the adenine‐only group, LB‐P22 supplementation significantly suppressed this upregulation at 10^9^ CFU for both genes, whereas 10^8^ CFU did not reach statistical significance (Figure 4A,B). Conversely, adenine exposure markedly reduced the expression of the mitochondrial fusion–related genes *Opa1* and *Mfn2*. LB‐P22 significantly increased *Opa1* transcript levels at both doses and *Mfn2* at 10^9^ CFU only; in both cases the levels remained below those of control kidneys, indicating partial rather than complete normalization (Figure 4C,D). These findings indicate that LB‐P22 reverses the transcriptional imbalance favouring mitochondrial fission in adenine‐induced renal injury. Consistent with these transcriptional changes, immunoblot analysis revealed that adenine‐induced kidney injury significantly increased Drp1 phosphorylation, a critical activation step for mitochondrial fission, and elevated MFF protein expression, while concomitantly reducing the levels of the mitochondrial fusion proteins OPA1 and Mfn2. Compared with the adenine‐only group, LB‐P22 at 10^9^ CFU significantly attenuated Drp1 phosphorylation and MFF expression and restored OPA1 levels, whereas at 10^8^ CFU only Mfn2 reached statistical significance (Figure 4E).

**FIGURE 4:**
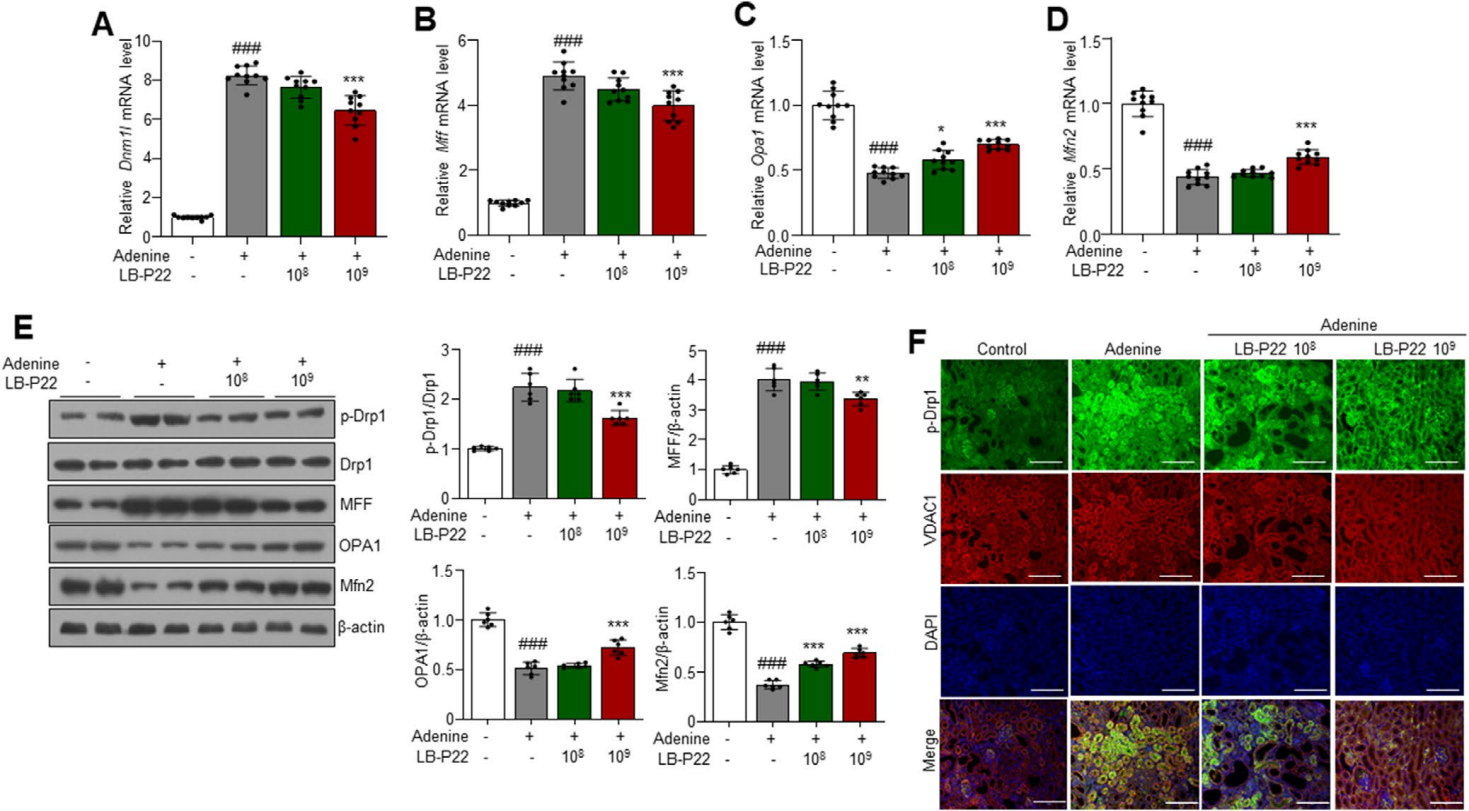
LB‐P22 suppresses Drp1‐mediated aberrant mitochondrial fission in adenine‐induced kidney injury. (A, B) Relative mRNA expression levels of the mitochondrial fission–associated genes *Dnm1l* (Drp1) and *Mff* in tissues, as determined by quantitative real‐time PCR. (C, D) Relative mRNA expression levels of the mitochondrial fusion–associated genes *Opa1* and *Mfn2* in tissues. (E) Representative immunoblot images showing phosphorylation of Drp1 (p‐Drp1), total Drp1, and mitochondrial dynamics–related proteins (MFF, OPA1, and Mfn2) in tissues from each experimental group, with the corresponding densitometric quantification. β‐Actin was used as a loading control. (F) Representative immunofluorescence images showing the subcellular localization of phosphorylated Drp1 (p‐Drp1) and mitochondrial distribution in renal tissues. p‐Drp1 is shown in green, mitochondria are labelled with VDAC1 (red), and nuclei are counterstained with DAPI (blue). Merged images highlight the mitochondrial recruitment of p‐Drp1. These images are representative, and colocalization was not quantified. Scale bar = 150 μm. Data are presented as the mean ± SD (*n* = 10 mice per group for panels A–D and *n* = 6 mice per group for panel E). Statistical significance was assessed by one‐way analysis of variance (ANOVA), followed by Tukey's multiple comparisons test. ^###^ *p* < 0.001 versus the control group; *p < 0.05, **p < 0.01, and ***p < 0.001 versus the adenine‐treated group.

To further validate the inhibitory effect of LB‐P22 on pathological mitochondrial fission in vivo, we examined the subcellular localization of phosphorylated Drp1 (p‐Drp1) in renal tissues by immunofluorescence staining. In control kidneys, p‐Drp1 exhibited a diffuse cytosolic distribution, whereas adenine administration induced pronounced p‐Drp1 accumulation in mitochondria, as indicated by increased colocalization with the mitochondrial marker VDAC1. LB‐P22 treatment visibly reduced adenine‐induced mitochondrial p‐Drp1 recruitment, restoring a distribution pattern closer to that observed in control kidneys; these images are representative and colocalization was not quantified (Figure 4F). Collectively, these results indicate that adenine‐induced kidney injury disrupts mitochondrial fission–fusion homeostasis by promoting aberrant Drp1 activation and mitochondrial recruitment, whereas LB‐P22 supplementation suppresses Drp1‐mediated pathological mitochondrial fission and restores mitochondrial dynamic balance in the injured kidney.

### 3.5 LB‐P22 Preserves Mitochondrial Morphology and Content and Reduces Mitochondrial Oxidative Stress in Adenine‐Induced Kidney Injury

To investigate how LB‐P22 protects against mitochondrial dysfunction in adenine‐induced CKD, we assessed PGC‐1α, a pivotal regulator of mitochondrial biogenesis and function [33, 34]. *Ppargc1a* (PGC‐1α) mRNA expression was significantly downregulated in adenine‐fed mice, whereas neither dose of LB‐P22 produced a significant change; the 10^9^ CFU group showed only a non‐significant trend toward higher expression (Figure 5A). PGC‐1α immunoreactivity appeared higher in LB‐P22‐treated kidneys by immunofluorescence (Figure 5B) and immunohistochemistry (Figure 5C), although these images are representative and were not quantified.

**FIGURE 5:**
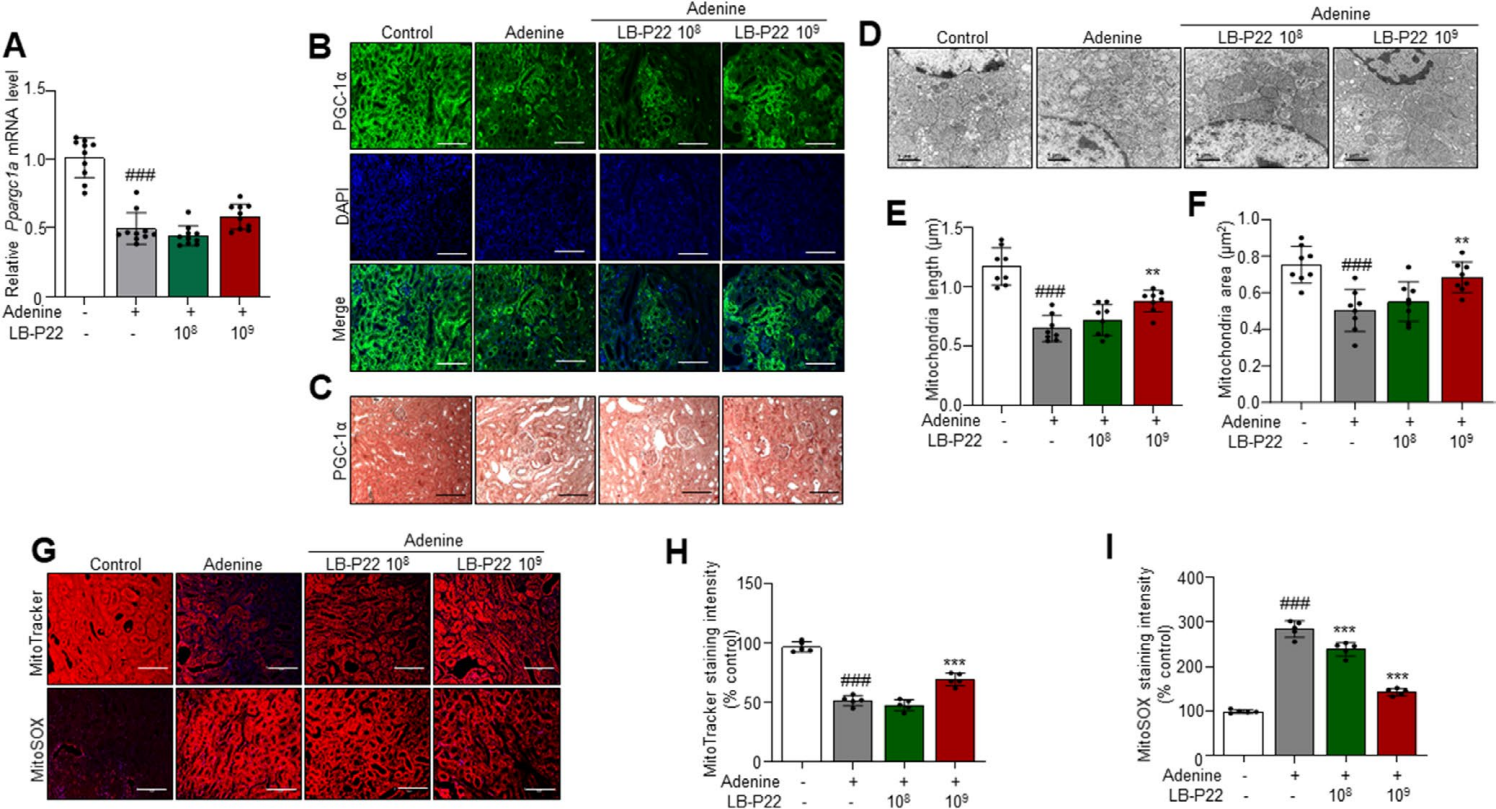
LB‐P22 preserves mitochondrial structure and function in mice with adenine‐induced CKD. (A) Expression of *Ppargc1a* mRNA, the transcript corresponding to peroxisome proliferator‐activated receptor γ coactivator‐1α (PGC‐1α). Values represent mean ± SD (*n* = 10 per group). (B) Immunofluorescence staining for PGC‐1α (green) with nuclear counterstaining using DAPI (blue). Scale bar = 150 μm. Representative images selected from five mice per group; shown for qualitative comparison and not quantified. (C) Immunohistochemical staining for PGC‐1α in renal tissues. Scale bar = 150 μm. Representative images selected from five mice per group; shown for qualitative comparison and not quantified. (D) TEM images showing mitochondrial ultrastructure in tubular epithelial cells; adenine‐fed mice exhibited swollen mitochondria and disrupted cristae, ameliorated by LB‐P22. Scale bar = 1 μm. (E, F) Quantification of mitochondrial length (E) and area (F). (G) Fluorescent micrographs of MitoTracker Deep Red and MitoSOX staining. Scale bar = 150 μm. (H, I) Quantification of MitoTracker (H) and MitoSOX (I) fluorescence intensity. Data are presented as the mean ± SD (*n* = 10 mice per group for panel A; *n* = 5 mice per group for panels G–I; and eight randomly selected regions from one representative micrograph per group for panels E and F). Statistical significance was assessed by one‐way analysis of variance (ANOVA), followed by Tukey's multiple comparisons test. ^###^ *p* < 0.001 versus the control group and **p < 0.01 and ***p < 0.001 versus the adenine‐treated group.

Mitochondrial ultrastructure was next examined by transmission electron microscopy. Renal tubular mitochondria in the adenine group exhibited pronounced structural damage, including swelling and disorganized cristae, whereas LB‐P22 at 10^9^ CFU partially preserved mitochondrial morphology (Figure 5D). Morphometric analysis confirmed this observation: compared with the adenine‐only group, both mitochondrial length (Figure 5E) and area (Figure 5F) were significantly increased at 10^9^ CFU, whereas 10^8^ CFU did not reach statistical significance.

Mitochondrial content and oxidative stress were then evaluated by MitoTracker Deep Red and MitoSOX staining, respectively (Figure 5G). Adenine treatment significantly reduced mitochondrial content and increased mitochondrial superoxide levels. Compared with the adenine‐only group, LB‐P22 significantly increased mitochondrial content at 10^9^ CFU (Figure 5H) and reduced mitochondrial superoxide at both 10^8^ and 10^9^ CFU (Figure 5I). Taken together, these results indicate that LB‐P22 preserves mitochondrial morphology and content and mitigates mitochondrial oxidative stress in adenine‐induced kidney injury, with the effects observed consistently at the higher dose.

### 3.6 LB‐P22 Mitigates Renal Inflammation and Oxidative Stress in Adenine‐Induced Kidney Injury

Renal fibrosis is primarily driven by chronic inflammation and oxidative stress, which represent key pathogenic mechanisms in chronic kidney disease [35, 36]. In an adenine‐induced CKD mouse model, LB‐P22 administration significantly attenuated renal inflammatory responses, as evidenced by significant reductions in the mRNA expression of pro‐inflammatory cytokines, including *Il1b*, *Il6*, and *Tnf*, which were elevated in adenine‐fed mice but substantially suppressed in the LB‐P22 (10^9^ CFU)–treated group (Figure 6A–C). In parallel, LB‐P22 significantly alleviated oxidative stress by restoring renal antioxidant defences, demonstrated by increased SOD, catalase, and GPx activities and reduced MDA at 10^9^ CFU; among these, catalase activity was also significantly increased at 10^8^ CFU (Figure 6D–G). Consistent with these in vivo findings, in vitro experiments using HEK293 cells supported the anti‐inflammatory potential of LB‐P22. In a preliminary viability screen under indoxyl sulfate (IS)–induced stress, LB‐P22 conditioned medium (CM) was tested at 0.1%–2%; 0.5% and 1% were selected for subsequent experiments as concentrations that did not further compromise viability, whereas 0.1% significantly reduced it (Figure S2A). Compared with IS alone, 1% CM produced a modest but significant increase in cell viability, whereas 0.5% and 2% did not differ significantly from IS (Figure S2A). IS markedly induced *Tnf* and *Il6* mRNA expression; CM significantly reduced *Tnf* at both 0.5% and 1%, and Il6 at 1% only (Figure S2B,C). Collectively, these results indicate that LB‐P22 mitigates inflammation and oxidative stress at both tissue and cellular levels, highlighting its protective role against CKD‐associated inflammatory and oxidative injury.

**FIGURE 6:**
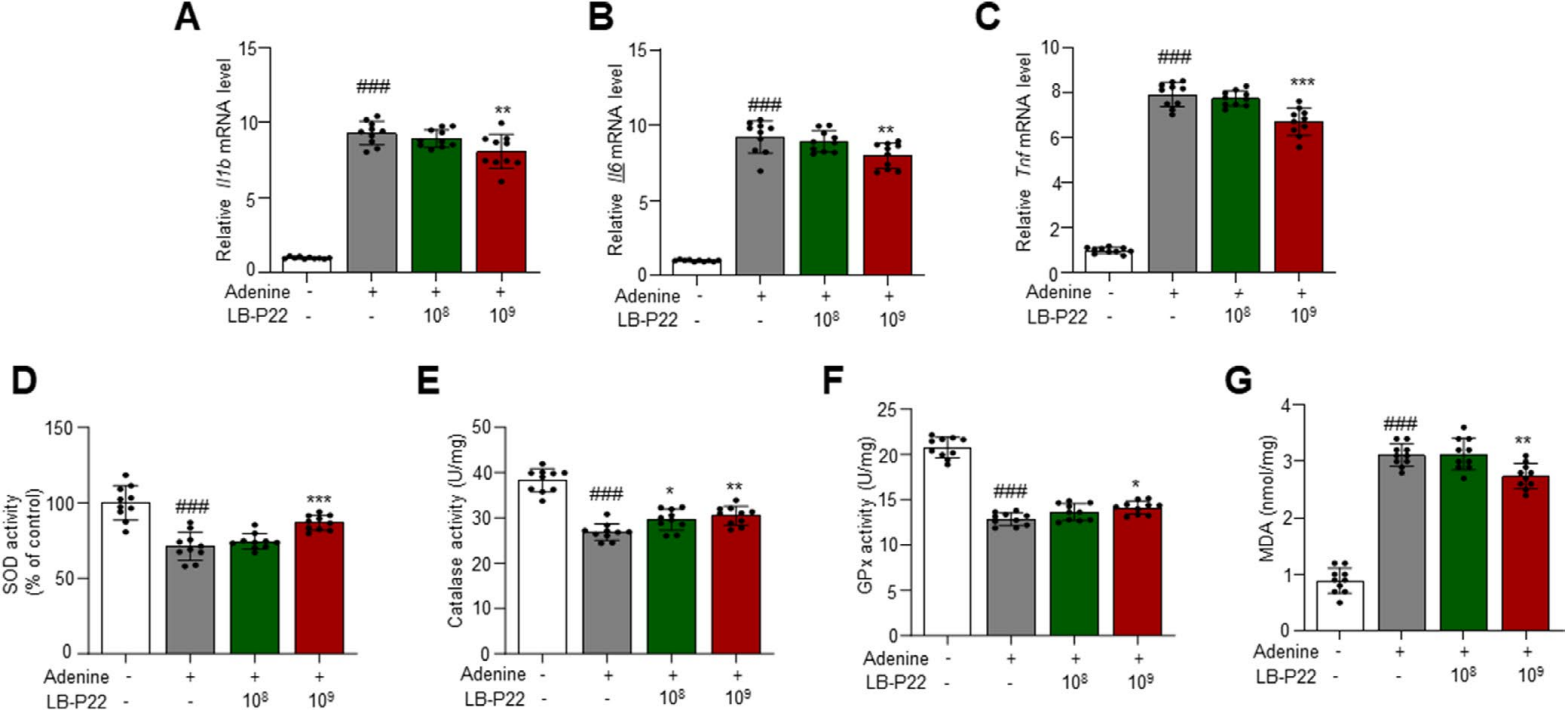
LB‐P22 alleviates inflammation and redox imbalance in mice with adenine‐induced CKD. (A–C) The mRNA profiles of *Il1b*, *Il6*, and *Tnf*, which correspond to IL‐1β, IL‐6, and TNF‐α, were evaluated in kidney tissues. (D–F) Renal tissues were analysed for the enzymatic activities of major antioxidants, including superoxide dismutase (SOD), catalase, and glutathione peroxidase (GPx). (G) Levels of malondialdehyde (MDA) in renal tissues, reflecting lipid peroxidation, were determined as a measure of oxidative stress. Data are presented as the mean ± SD (10 animals per group). Statistical significance was assessed by one‐way analysis of variance (ANOVA), followed by Tukey's multiple comparisons test. ^###^ *p* < 0.001 versus the control group and *p < 0.05, **p < 0.01, and ***p < 0.001 versus the adenine‐treated group.

### 3.7 LB‐P22 Inhibits TGF‐β1/MAPK Pathway Activation in Adenine‐Induced Kidney Injury

TGF‐β1 is a well‐established master regulator of renal fibrosis, orchestrating fibrotic remodelling through its downstream signalling pathways [37]. Among these, the MAPK pathway, particularly ERK, JNK, and p38 signalling, drives epithelial‐to‐mesenchymal transition and tissue scarring under chronic kidney injury conditions [38, 39]. Immunoblot analysis showed that TGF‐β1 protein was markedly upregulated in the kidneys of adenine‐fed mice, confirming activation of profibrotic signalling; compared with the adenine‐only group, TGF‐β1 expression was significantly reduced at 10^9^ CFU, whereas 10^8^ CFU did not reach statistical significance (Figure 7A). Consistent with this finding, immunofluorescence staining showed marked accumulation of TGF‐β1 in the tubular epithelial compartments of adenine‐fed mice, which appeared attenuated in LB‐P22–treated kidneys; these images are representative and were not quantified (Figure 7B). To explore the mechanistic basis of this modulation, we assessed the phosphorylation status of MAPK components. Phosphorylation of JNK, ERK, and p38 was significantly increased in the adenine‐fed group, consistent with activation of the MAPK pathway. Compared with the adenine‐only group, LB‐P22 significantly reduced the phosphorylation of all three kinases at 10^9^ CFU, whereas no significant change was observed at 10^8^ CFU (Figure 7C). Collectively, these results indicate that LB‐P22 exerts its anti‐fibrotic activity predominantly at the higher dose through regulation of the TGF‐β1/MAPK pathway.

**FIGURE 7:**
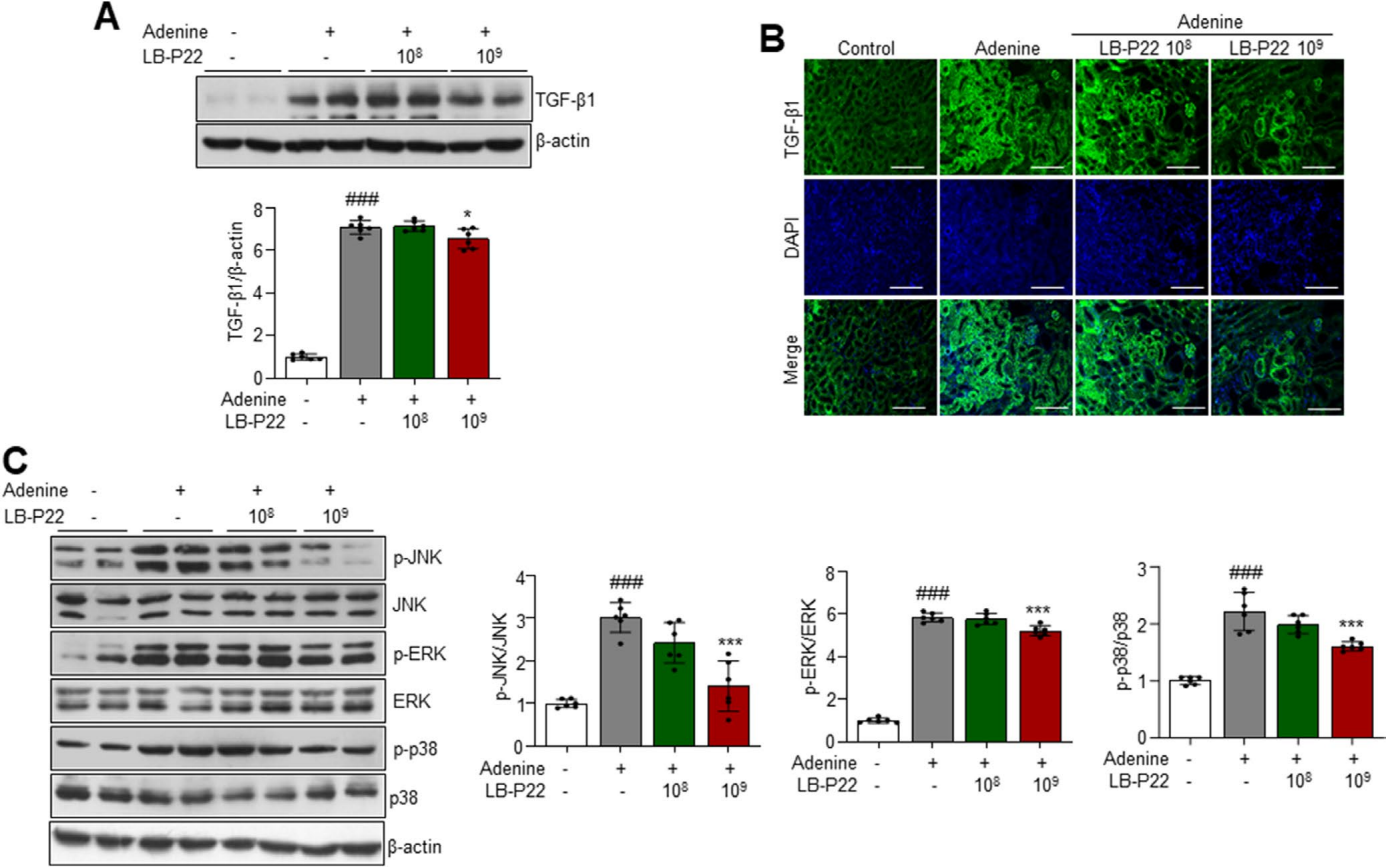
LB‐P22 modulates TGF‐β1/MAPK pathways in mice with adenine‐induced CKD. (A) Immunoblot analysis of TGF‐β1 expression in renal tissues with corresponding quantification. (B) Immunofluorescence images showing renal expression of TGF‐β1 (green) with nuclear DAPI counterstaining (blue). These images are representative and were not quantified. Scale bar = 150 μm. (C) The expression levels of total and phosphorylated forms of MAPK pathway proteins, including ERK1/2, p38, and JNK by immunoblotting and its quantification. Data are presented as the mean ± SD (*n* = 6 animals per group). Statistical significance was assessed by one‐way analysis of variance (ANOVA), followed by Tukey's multiple comparisons test. ^###^ *p* < 0.001 versus the control group; *p < 0.05, and ***p < 0.001 versus the adenine‐treated group.

## 4 Discussion

The present study indicates that *Lactococcus lactis* subsp. *hordniae* LB‐P22 protects against renal fibrosis and functional decline in an adenine‐induced chronic kidney disease model. LB‐P22 attenuated tubular injury and interstitial fibrosis, reduced the gut‐derived uremic toxin indoxyl sulfate, and suppressed Nox4‐driven oxidative stress. These effects were associated with restoration of PDIA1–Drp1 redox regulation, preservation of mitochondrial dynamics, and attenuation of profibrotic and inflammatory signalling. Together, our findings identify LB‐P22 as a probiotic modulator of the gut–kidney–mitochondria axis with therapeutic potential in chronic kidney disease. The inclusion of two doses allowed comparison across the range tested. Statistically significant effects were observed almost exclusively at 10^9^ CFU, and direct comparison between the two doses showed significantly greater preservation of body weight at 10^9^ than at 10^8^ CFU, supporting a dose‐dependent renoprotective action of LB‐P22. This study provides, to our knowledge, the first evidence that *Lactococcus lactis* subsp. *hordniae* LB‐P22 protects against renal fibrosis in an adenine‐induced chronic kidney disease model (Figure 1A–E). As expected, adenine administration induced severe renal dysfunction and fibrotic remodelling [40, 41], whereas LB‐P22 treatment significantly attenuated renal injury and suppressed key fibrotic markers, including α‐smooth muscle actin and type I collagen.

Accumulating evidence underscores the role of the gut–kidney axis in CKD progression through the production of gut‐derived uremic toxins [42, 43]. In adenine‐induced CKD, circulating levels of indoxyl sulfate (IS), phenyl sulfate, and p‐cresyl sulfate are elevated and contribute to oxidative stress, inflammation, and fibrosis [44, 45]. In this study, LB‐P22 selectively reduced serum IS without affecting pCS, suggesting targeted modulation of microbial metabolism rather than global suppression of uremic toxins. This finding is consistent with emerging evidence that selective regulation of gut microbial metabolic pathways can confer renoprotective effects [46, 47].

Oxidative stress and mitochondrial dysfunction are key drivers of chronic kidney disease, promoting tubular injury and fibrotic remodelling [48, 49]. Among the major sources of pathological reactive oxygen species (ROS) in the kidney, NADPH oxidase 4 (Nox4) has been shown to play a central role and is consistently upregulated in both experimental models and patients with CKD [50, 51]. In line with these observations, adenine administration increased NADPH oxidase activity, Nox4 expression, and intracellular ROS levels, whereas LB‐P22 at 10^9^ CFU significantly reduced NADPH oxidase activity and Nox4 expression (Figure 3A,B), with a corresponding decrease in CellROX staining intensity (Figure 3C). These results suggest that suppression of Nox4‐dependent oxidative signalling contributes to the renoprotective effects of LB‐P22 in adenine‐induced CKD.

A key novel finding of this study is the identification of protein disulfide isomerase A1 (PDIA1) as a redox‐sensitive target in adenine‐induced chronic kidney disease. PDIA1 plays a central role in cellular redox homeostasis through thiol–disulfide exchange reactions that regulate protein redox balance and stress responses [52, 53, 54]. Previous studies have shown that reduced PDIA1 expression or activity contributes to oxidative stress–associated tissue injury in cardiovascular and metabolic disorders [28, 55]. In line with these observations, we found that adenine‐induced renal injury was accompanied by a pronounced decrease in PDIA1 abundance and enzymatic activity, indicating disruption of PDIA1‐dependent redox regulation in the kidney (Figure 3D,E). Importantly, LB‐P22 supplementation significantly increased PDIA1 expression and activity, suggesting that preservation of PDIA1 redox capacity represents a key mechanism through which LB‐P22 maintains redox balance under CKD conditions.

Disruption of PDIA1‐dependent redox regulation in adenine‐induced CKD was associated with aberrant activation of dynamin‐related protein 1 (Drp1), a key mediator of mitochondrial fission. Redox‐dependent Drp1 modifications, including sulfenylation and phosphorylation, are known to promote pathological mitochondrial fragmentation [56, 57]. Consistent with this, adenine‐induced renal injury increased Drp1 oxidative modification and fission‐related signalling, whereas LB‐P22 supplementation attenuated Drp1 activation and suppressed aberrant mitochondrial fission, preserving mitochondrial morphology (Figure 5D–F). Together, these findings extend previous work by implicating disruption of the PDIA1–Drp1 redox regulatory axis as a key mechanism underlying mitochondrial dysfunction in CKD.

Mitochondrial dysfunction is a central driver of renal fibrosis in chronic kidney disease, as mitochondria regulate not only energy metabolism but also redox signalling, cell survival, and inflammatory responses [58]. In this study, LB‐P22 preserved mitochondrial morphology and content and reduced mitochondrial ROS, accompanied by enhanced antioxidant defences and reduced lipid peroxidation (Figures 5 and 6); PGC‐1α expression showed a non‐significant trend toward recovery. Consistent with previous reports, preservation of mitochondrial function and redox balance limits fibrotic progression in experimental kidney disease models [59, 60], supporting a mechanistic link between mitochondrial protection and the anti‐fibrotic effects observed with LB‐P22.

Renal fibrosis is driven in part by persistent activation of profibrotic signalling pathways, particularly the TGF‐β1/MAPK axis. In adenine‐induced CKD, accumulation of 2,8‐dihydroxyadenine activates TGF‐β1 signalling and downstream MAPK cascades, including ERK, JNK, and p38, thereby promoting tubulointerstitial fibrosis [61, 62, 63]. In this study, LB‐P22 significantly suppressed ERK, JNK, and p38 phosphorylation, indicating attenuation of MAPK‐dependent profibrotic signalling (Figure 7). Given the established crosstalk between MAPK activation and mitochondrial dysfunction [62], the ability of LB‐P22 to concurrently modulate MAPK activity and mitochondrial integrity highlights a coordinated mechanism underlying its anti‐fibrotic effects.

In conclusion, this study indicates that *Lactococcus lactis* subsp. *hordniae* LB‐P22 confers renoprotective effects that are associated with reduced uremic toxin levels and improved mitochondrial redox homeostasis. Beyond conventional probiotic actions, LB‐P22 selectively reduced indoxyl sulfate and restored PDIA1‐dependent regulation of Drp1, thereby preserving mitochondrial integrity and suppressing fibrotic signalling. These findings provide mechanistic evidence that probiotic intervention can influence intracellular redox signalling pathways, representing a conceptual advance from microbiome modulation toward organelle‐targeted therapeutics. Collectively, our results position LB‐P22 as a novel modulator of the gut–kidney–mitochondria axis and support its translational potential as a microbiome‐based strategy for slowing CKD progression.

Because the involvement of PDIA1 and gut microbial metabolism was inferred from associated changes rather than from direct causal testing or microbiota analysis, these mechanistic links warrant further confirmation. Future studies should investigate whether LB‐P22–mediated regulation of PDIA1 and mitochondrial dynamics is reproducible in human CKD and whether circulating indoxyl sulfate may serve as a predictive biomarker for probiotic responsiveness. Clinical validation will be essential to determine whether targeting mitochondrial redox signalling through microbiome modulation can translate into disease‐modifying therapy.

## Author Contributions

**Hwa‐Young Lee:** conceptualization, writing – original draft, methodology, investigation. **Mikyung Song:** conceptualization, investigation, writing – original draft. **Do‐Sung Kim:** conceptualization, investigation, methodology, validation. **Myoung Ja Chung:** conceptualization, investigation, validation. **Hyewon Lee:** writing – review and editing, data curation, formal analysis, validation. **Geum‐Hwa Lee:** conceptualization, data curation, investigation, visualization. **Han‐Jung Chae:** writing – original draft, supervision, validation, writing – review and editing. **Jaeseok Shim:** conceptualization, writing – original draft, supervision. **Hwangeui Cho:** conceptualization, writing – original draft, investigation, supervision.

## Funding

This research was supported by the National Research Foundation of Korea (NRF‐2021R1C1C2007371, RS‐2024‐00335573). This work was supported by “Food Functionality Evaluation program” under the Ministry of Agriculture, Food and Rural Affairs.

## Conflicts of Interest

The authors declare no conflicts of interest.

## Supporting information


**Supplementary Figure 1** *Lactococcus lactis* subsp. *hordniae* LB‐P22 administration does not cause systemic toxicity in mice with adenine‐induced chronic kidney disease. (A) Illustration of the experimental procedure. To evaluate the therapeutic efficacy of *Lactococcus lactis* subsp. *hordniae* LB‐P22 (LB‐P22), mice were orally administered LB‐P22 at doses of 10^8^ or 10^9^ CFU for 1 week in advance of CKD induction, followed by a 3‐week coadministration of 0.75% adenine and LB‐P22. Following completion of the treatment period, mice were euthanized for biochemical and histopathological analyses. (B) Body weight was monitored throughout the 4‐week treatment. (C, D) Serum activities of glutamic oxaloacetic transaminase (GOT) and glutamic pyruvic transaminase (GPT) were measured to assess hepatotoxicity. Data are presented as the mean ± SD (*n* = 10 animals per group). Statistical significance was assessed by one‐way analysis of variance (ANOVA), followed by Tukey's multiple comparisons test. ^###^ *p* < 0.001 versus the control group; *p < 0.05 and ***p < 0.001 versus the adenine‐treated group.
**Supplementary Figure 2**. LB‐P22 treatment inhibited cytotoxicity induced by IS in HEK293 cells and mRNA expression of inflammation cytokines. (A) Cells were treated for 24 h with LB‐P22 conditioned medium (CM) at 0.1%, 0.5%, 1%, or 2% in the presence or absence of 5 mM IS. Cell viability was determined by WST‐8 assay. (B, C) The mRNA expression of the *TNF* and *IL6*. Data are presented as the mean ± SD (*n* = 3 per group). Statistical significance was assessed by one‐way analysis of variance (ANOVA), followed by Tukey's multiple comparisons test. ^###^ *p* < 0.001 and ^####^ *p* < 0.0001 versus the control group; **p < 0.01, ***p < 0.001, and ****p < 0.0001 versus the IS group.

## Data Availability

Data available on request from the authors.
