# Supplementary material for: PDI‐Mediated Drp1 Redox Regulation Preserves Mitochondrial Function and Suppresses Fibrosis in Adenine‐Induced Kidney Injury by Lactococcus lactis subsp. hordniae LB‐P22
Source: J Cell Mol Med. 2026 Nov 13;30(22):e71456. doi: 10.1111/jcmm.71456 (PMC13768801; doi:10.1111/jcmm.71456)
Supplement: Supplementary file 1 [file JCMM-30-e71456-s001.docx]

**PDI-Mediated Drp1 Redox Regulation Preserves Mitochondrial Function and Suppresses Fibrosis in Adenine-Induced Kidney Injury by *Lactococcus lactis* subsp. *hordniae* LB-P22**

Hwa-Young Lee^1,2^, Mikyung Song^3^, Do-Sung Kim^1,2^, Myoung Ja Chung^4^, Hyewon Lee^5^, Geum-Hwa Lee^2^, Han-Jung Chae^1, 2, 5*^, Jaeseok Shim^3*^, Hwangeui Cho^5*^

^1^Non-Clinical Evaluation Center Biomedical Research Institute, Jeonbuk National University Hospital, Jeonju, Jeonbuk 54907, Republic of Korea; ^2^Research Institute of Clinical Medicine of Jeonbuk National University-Biomedical Research Institute of Jeonbuk National University Hospital, Jeonju, Jeonbuk 54907, Republic of Korea; ^3^R&D Center, LISCure Bio Inc., Seongnam 13488, Republic of Korea; ^4^Department of Pathology, Jeonbuk National University Medical School, Jeonju, Jeonbuk 54907, Republic of Korea; ^5^School of Pharmacy, Jeonbuk National University, Jeonju, Jeonbuk 54896, Republic of Korea

^*^ Corresponding authors: Han-Jung Chae, School of Pharmacy, Jeonbuk National University, Jeonju, Jeonbuk 54896, Republic of Korea; Non-Clinical Evaluation Center Biomedical Research Institute, Jeonbuk National University Hospital, Jeonju, Jeonbuk 54907, Republic of Korea, Tel: 82-63-270-3092, Fax: 82-63-275-2855, E-mail: [hjchae@jbnu.ac.kr](mailto:hjchae@jbnu.ac.kr); Jaeseok Shim, R&D Center, LISCure Bio Inc., Seongnam 13488, Republic of Korea, E-mail: [jsshim@liscure.bio](mailto:jsshim@liscure.bio); Hwangeui Cho, School of Pharmacy, Jeonbuk National University, Jeonju, Jeonbuk 54896, Republic of Korea, E-mail: hecho@jbnu.ac.kr

**Supplementary Figure 1. *Lactococcus lactis* subsp. *hordniae* LB-P22 administration does not cause systemic toxicity in mice with adenine-induced chronic kidney disease.** (A) Illustration of the experimental procedure. To evaluate the therapeutic efficacy of *Lactococcus lactis* subsp. *hordniae* LB-P22 (LB-P22), mice were orally administered LB-P22 at doses of 10⁸ or 10⁹ CFU for 1 week in advance of CKD induction, followed by a 3-week coadministration of 0.75% adenine and LB-P22. Following completion of the treatment period, mice were euthanized for biochemical and histopathological analyses. (B) Body weight was monitored throughout the 4-week treatment. (C, D) Serum activities of glutamic oxaloacetic transaminase (GOT) and glutamic pyruvic transaminase (GPT) were measured to assess hepatotoxicity. Data are presented as the mean ± SD (n = 10 animals per group). Statistical significance was assessed by one-way analysis of variance (ANOVA), followed by Tukey’s multiple comparisons test. ^###^p < 0.001 versus the control group; ^*^*p* < 0.05 and ^***^*p* < 0.001 versus the adenine-treated group.

**Supplementary Figure 2. LB-P22 treatment inhibited cytotoxicity induced by IS in HEK293 cells and mRNA expression of inflammation cytokines**. (A) Cells were treated for 24 h with LB-P22 conditioned medium (CM) at 0.1, 0.5, 1, or 2% in the presence or absence of 5 mM IS. Cell viability was determined by WST-8 assay. (B-C) The mRNA expression of the *TNF* and *IL6*. Data are presented as the mean ± SD (n = 3 per group). Statistical significance was assessed by one-way analysis of variance (ANOVA), followed by Tukey’s multiple comparisons test. ^###^*p* < 0.001 and ^####^*p* < 0.0001 versus the control group; ^**^*p* < 0.01, ^***^*p* < 0.001, and ^****^*p* < 0.0001 versus the IS group.
